# Surface Functionalization of Poly(lactic acid) via Deposition of Hydroxyapatite Monolayers for Biomedical Applications

**DOI:** 10.1021/acs.langmuir.3c01914

**Published:** 2023-10-26

**Authors:** Katarzyna Dopierała, Monika Knitter, Monika Dobrzyńska-Mizera, Jacek Andrzejewski, Aneta Bartkowska, Krystyna Prochaska

**Affiliations:** † Institute of Chemical Technology and Engineering, Poznan University of Technology, Berdychowo 4, 60-965 Poznań, Poland; ‡ Institute of Material Technology, Poznan University of Technology, Piotrowo 3, 61-138 Poznań, Poland; § Poznan University of Technology, Faculty of Materials Engineering and Technical Physics, Institute of Material Science and Engineering, Jana Pawła II 24, 61-138 Poznań, Poland

## Abstract

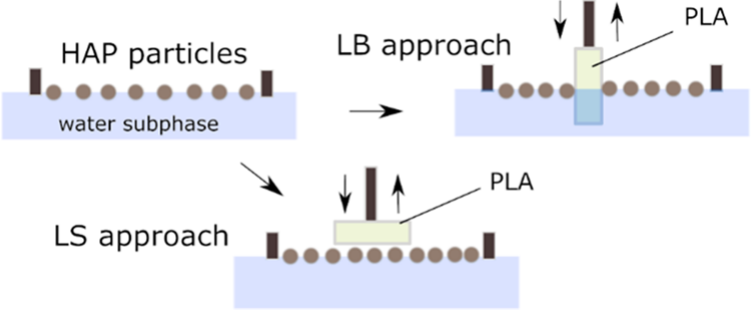

The surface modification of poly(lactic acid) (PLA) using hydroxyapatite (HAP) particles via Langmuir–Blodgett (LB) and Langmuir–Schaefer (LS) approaches has been reported. The HAP monolayer was characterized at the air/water interface and deposited on three-dimensional (3D) printed poly(lactic acid). The deposition of HAP particles using the LS approach led to a larger surface coverage in comparison to the LB method, which produces a less uniform coating because of the aggregation of the particles. After the transfer of HAP on the PLA surface, the wettability values remained within the desired range. The presence of HAP on the surface of the polymer altered the topography and roughness in the nanoscale, as evidenced by the atomic force microscopy (AFM) images. This effect can be beneficial for the osteointegration of polymeric implants at an early stage, as well as for the reduction of the adherence of the microbial biofilm. Overall, the results suggest that the LS technique could be a promising approach for surface modification of PLA by hydroxyapatite with respective advantages in the biomedical field.

## Introduction

The recent advancements in tissue engineering, especially in the field of bone regeneration, force further improvements in material engineering in order to fulfill patients’ needs. For these purposes, various types of bioresorbable polymers and copolymers of lactic acid, ethane-1,2-diol (ethyl glycol), or chitosan (poly(d)-glucosamine) are commonly used.^1−5^ Polylactides (PLAs) are notably interesting due to their bioresorbability while maintaining the appropriate mechanical strength and easy processing via basic industrial methods. Their physicochemical and mechanical properties may be further tailored by bulk modification with bioactive inorganic fillers, which alter degradation kinetics, resorption, and mechanical properties.^6,7^ However, apart from bulk characteristics, it is essential to consider PLA surface properties in order to enhance the interactions with cells. The biological response to the biomaterial implanted into the human body involves several steps, namely, hydration by water molecules from blood or extracellular fluids followed by the adsorption of various proteins and reactions between the biomaterial and tissue. The goals of surface modification include changes in wettability, surface free energy, topography, and adhesiveness.^8,9^ These changes result in a decreased level of biofilm formation, a critical problem in bone regeneration. The most important obstacles in PLA–cell interactions are the hydrophobicity of the polymer and the lack of specific functional groups to promote cell adhesion and growth on its surface. Surface modification of PLA is possible using air plasma treatment,^10^ ion or electron beams,^11^ or coating techniques.^12^ Altering the surface properties of a biomaterial to enhance its performance in a biological environment should retain the bulk properties of the polymer. It means that the modified zone at the surface should be as thin as possible.

Therefore, the Langmuir–Blodgett technique (LB) is one of the physical methods to modify the surface properties of a polymeric material.^13−15^ Low-dimensional LB films have been studied for years.^16^ Several publications have appeared recently documenting LB films built of gold nanoparticles,^17^ zinc oxide,^18,19^ magnetic nanoparticles and nanowires,^20,21^ fullerenes,^22^ graphene, and graphene oxide.^23,24^ Ariga introduced the concept of nanoarchitectonics, which uses a liquid interface to form controlled nanostructures for nanosensing, nanophotonics, and molecular electronics.^25^ In addition, much research on complex, multicomponent monolayers has been done including thermodynamic characterization, rheological studies, and deposition on various substrates.^26−28^ For instance, various biomolecules can be incorporated into the lipid monolayer such as proteins,^29,30^ chitosan,^31^ collagen,^32^ and keratin as well as xenobiotics,^33,34^ extending the applications of Langmuir films in the biomedical field.

It was shown that LB coatings deposited on a polymeric or metallic substrate may improve osteoblast alignment and/or biomineralization.^35,36^ Hence, the application of LB films is versatile^37−40^ with the greatest advantages being the simplicity of the method and possible upscaling. A modification of the LB method for the fabrication of high-quality films on a solid substrate was proposed by Langmuir and Schaefer.^41^ In the Langmuir–Schaefer (LS) approach, the substrate is lifted horizontally through the floating monolayer. This strategy is particularly designed for the deposition of rigid monolayers and protein films on a solid substrate. The LS technique was reported as useful in culturing stem cells,^42,43^ preparation of the bacterial antifouling surface,^44^ and immobilization of enzymes.^45^ Apart from versatility, LB and LS techniques offer controllable thickness of the film and the deposition at thermodynamically stable conditions.^46^ It can also be easily scaled up for larger areas.^47^

This work aims at the fabrication and characterization of hydroxyapatite Langmuir films and the assessment of their ability to be deposited on poly(lactic acid) as a selected biomaterial. To the best of our knowledge, such a system has not yet been presented in the literature. The paper details the formation of hydroxyapatite monolayers at the air/water interface, as well as their characterization after transfer onto poly(lactic acid) substrates via LB and LS methods.

## Materials and Methods

### Materials

A polylactide-based unmodified filament, with a diameter of 1.75 mm, supplied by PlastSpaw (Lubliniec, Poland) was utilized. Commercially available synthetic hydroxyapatite with a chemical formula of [Ca_5_(OH)(PO_4_)_3_]_*x*_, abbreviated HAP, was kindly supplied by Syntplant sp. z o.o. (Poland) in the form of whitish powder with a defined elemental composition (CaO 54.22, P_2_O_5_ 33.05, and MgO 0.11%m/m). Absolute ethanol and 2-propanol were used as solvents for the preparation of a monolayer experiment.

### Samples’ Production

The layer-by-layer (fused deposition modeling) technique was used to three-dimensional (3D) print the substrate models for further surface functionalization. The samples had a size of 15 mm × 15 mm × 1 mm and a single layer thickness of 0.2 mm. To ensure the samples’ geometric accuracy, an additional two-layer external shell was designed. Subsequently, PrusaSlicer software was used to generate the model machine code (g-code) and further utilized for 3D printing on the Prusa MK3 machine (from PrusaResearch, Czech Republic). The machine was equipped with a 0.4 in. brass nozzle and worked in the direct drive system. The bed table temperature was set at 60 °C, while the nozzle temperature was 215 °C. The models were printed with full infill (100%), and individual layers were intersected at an angle of 90 °C. The printing speed for perimeters was set at 50 mm/s, while the infill layers were prepared at 80 mm/s.

### Isotherm Studies

A Teflon Langmuir trough (KSV Nima) with a surface area of 273 cm^2^ was used for all monolayer experiments. Ultrapure water (18 MΩ·cm, pH 6.20, TOC 1–3 ppb) was prepared by a two-step purification process by a DEMIWA 5 filtration system and an ELGA PURELAB Classic UV and used as a subphase. The hydroxyapatite dispersions (1 mg/mL) were prepared in absolute ethanol and sonicated for 30 min before spreading at the air/water interface. To obtain a narrow HAP particle size range, the dispersion was further filtered through a syringe filter of 0.25 μm pore size. As a result, the particles with an average size of 200 ± 2.5 nm were used, as evidenced by the dynamic light scattering data. The monolayer was prepared by carefully spreading small droplets of the HAP dispersion on the water surface. After 10 min, the film was compressed at a rate of 10 mm/min. The surface pressure was measured by the Wilhelmy method using a platinum plate as a sensor. The accuracy and the resolution of measurements were 0.1 mN/m and 4 μN/m, respectively. The temperature of 21 ± 1 °C was kept constant during the monolayer experiment with a Julabo F12 thermostat. The results were recorded as the surface pressure (π) versus trough area (*A*). The compression modulus (*C*_s_^–1^) was determined from the isotherm data using eq 1
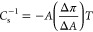
1in which *A* means the area and *T* is the temperature.

### Brewster Angle Microscopy (BAM)

The morphology of the films spread at the air/water interface was investigated using a microBAM (KSV Nima) coupled with a Langmuir trough. A black plate was immersed in a subphase to absorb the refracted beam. The camera had a field of view of 3.6 mm × 4.0 mm and worked at a resolution of approximately 6 μm per pixel. The images were captured during the compression of the film at various surface pressures.

### Dilational Viscoelasticity

The dilational viscoelasticity versus frequency (ν) was measured by means of oscillatory barrier experiments. The theoretical background of the method has been detailed elsewhere.^48^ In general, the experiment was based on the mechanical perturbation of the surface area, and the system response was measured. After the compression of the HAP film to a desired surface pressure, the sinusoidal deformations of interfacial area (*A*) were forced by the movement of the barriers at a frequency range of 0.03–0.08 Hz and an amplitude of 2%, which ensured a linear regime. The dilational response of the film is a complex dilational modulus, i.e., the function containing two components: the dilational elastic modulus (*E*′) and the storage (loss) modulus (*E*″), which characterize the solid-like and fluid-like contributions to the measured response.

### Deposition of Langmuir–Blodgett and Langmuir–Schaefer Films

PLA samples were cleaned with isopropyl alcohol before the deposition. For Langmuir–Blodgett deposition, the sample was held vertically to the subphase, while for the Langmuir–Schaefer method, the transfer was performed horizontally. All depositions were performed using the HAP monolayer compressed to π = 20 mN/m. If not stated otherwise, the deposition rate was 1 mm/min. In the case of the LB method, the sample was immersed into the subphase before spreading the HAP dispersion, and the deposition started with an upstroke. In the case of LS deposition, the substrate was lowered to touch the surface and withdrawn. After the deposition, the substrate was dried and further characterized. The quality of the transfer was described by the transfer ratio (TR) defined as the ratio of the decrease in a monolayer area during a deposition stroke to the area of the substrate.

### Atomic Force Microscopy (AFM)

Atomic force microscopy (AFM) was used to characterize the topography of the films transferred via the LB and LS approaches. The images were captured using an NX 10 apparatus (Park System, Korea). The microscope was operated in tapping mode using silicone cantilevers (All in One, Budget Sensors) having a force constant of 7.4 N/m. If not stated otherwise, all measurements were conducted at 20 ± 1 °C within 24 h from the deposition. The data extracted from AFM images allowed us to determine (1) mean roughness (*S*_a_), which characterizes the average deviation of all points’ roughness profile from a mean line over the evaluation length, (2) RMS roughness (*S*_q_), which is the root-mean-square average of the profile height deviations from the mean line recorded within the evaluation length, (3) skewness (*S*_sk_), which describes the degree of height distortion from the normal distribution, (4) excess kurtosis, which determines the intensity of extreme height values, (5) maximum height (*S*_*z*_), and (6) surface coverage.

### Scanning Electron Microscopy

Microstructure observations were carried out using a MIRA-3 scanning electron microscope (TESCAN, Brno, Czech Republic) on the surface of the specimens by using the secondary electrons (SE) as well as the backscattered electrons (BSE). Prior to the SEM observation, all specimens were covered with a thin layer of carbon. The sputtered layer is designed to reduce the effect of accumulation of electric charge as a result of the electron beam interaction with the specimen surface. For this purpose, a JEOL JEE 4B vacuum evaporator was used.

The chemical composition was investigated via energy-dispersive spectroscopy (EDS) using an UltimMax energy-dispersive spectrometer (Oxford Instruments, High Wycombe, U.K.). AZtec Energy Live Standard software was utilized to perform point analysis to evaluate elements’ content.

### Contact Angle Measurements

The water contact angle (WCA) was measured using a Theta Lite optical tensiometer (Biolin Scientific, Helsinki, Finland) working in a sessile drop mode. At least three water droplets of 3 μL each were placed on a sample using an automated dispenser, and the average contact angle was determined by One Attension software. The results were obtained for pure PLA and the substrate coated by HAP either via the LB or LS approach.

## Results and Discussion

**Figure 1:**
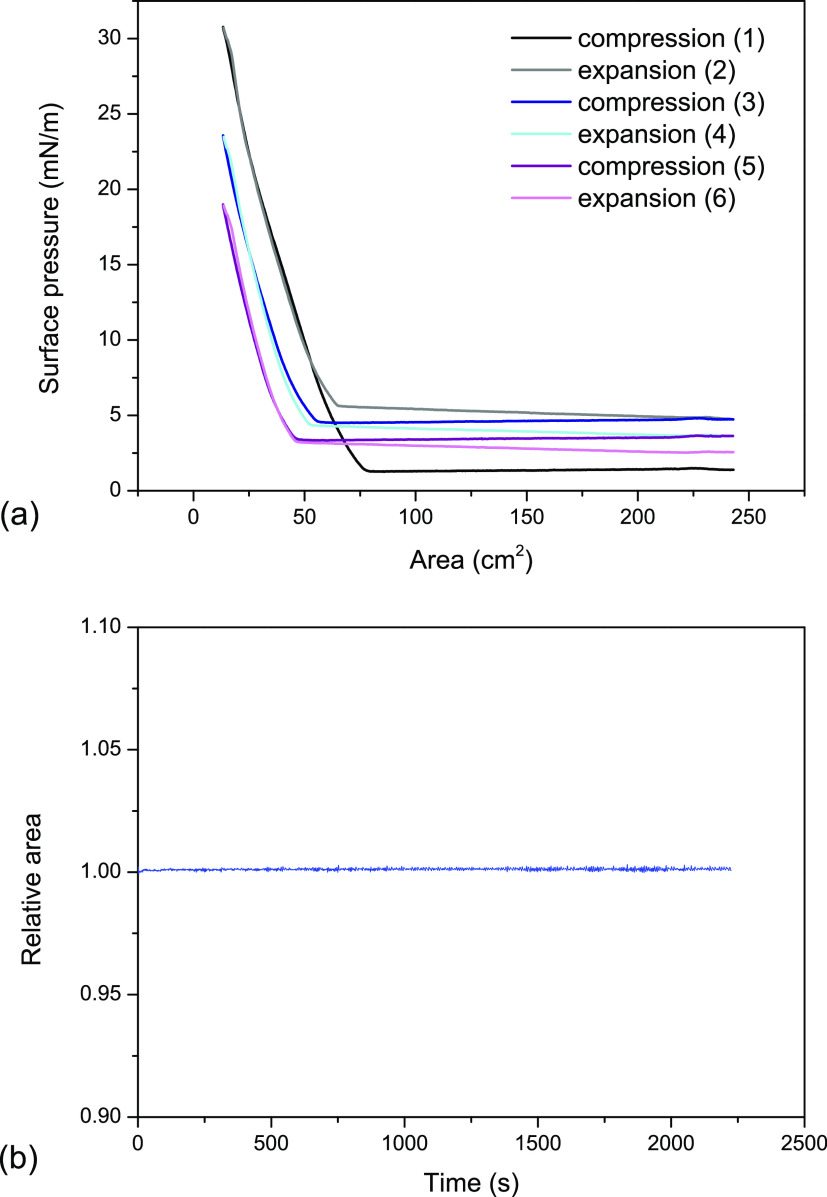
Surface pressure–area isotherms (a) recorded as compression–expansion cycles and the relaxation curve recorded after compression of the film to π = 20 mN/m (b).

The surface pressure–area isotherms of HAP particles spread at the air/water interface are presented in Figure 1a. The experiment was performed in three compression–expansion cycles. Within the first compression cycle, the surface pressure increased to 31 mN/m, wherein, upon the first film expansion, the surface pressure remained even higher in comparison to the initial value. The second compression caused the growth of the π value to 23 mN/m, while after the third one, the surface pressure reached 18 mN/m. These results indicate irreversible compression of the film, which might be explained by aggregation of the molecules at the interface or their desorption to the subphase. To verify this hypothesis in the relaxation experiment, the monolayer was compressed to π = 20 mN/m, and the barriers were forced to keep the surface pressure constant. The results shown in Figure 1b proved negligible changes in the relative area, indicating minor material loss at the interface; therefore, we concluded that the film was stable at the air/water interface. Due to the absence of an amphiphilic structure, the main forces responsible for the stability of nanoparticles in the monolayer are van der Waals attractions and steric repulsions.^49^ Hence, the hysteresis in Figure 1a may be attributed to the aggregation of the nanoparticles, causing limited mechanical stability under continuous compression and expansion.

The isotherm data allow us to calculate the compression modulus using eq 1. Figure S1 in the Supporting Information (SI) shows the compression modulus curves determined numerically and smoothed for the first, second, and third compression. The maximum value of *C*_s_^–1^ reaches 26 mN/m, which classifies the monolayer as a liquid-expanded film. Since the surface pressure in the isotherm is plotted against the trough area and the molecular area is not available, it must be remembered that the compression modulus for the HAP monolayer cannot be directly compared to the values determined for other monolayers. The mechanical properties of the HAP film alter with the following compressions; however, the physical state remains the same. The deposition of the monolayer on a solid substrate is usually preferred at the surface pressure corresponding to the solid state (*C*_s_^–1^ > 250 mN/m). Here, the transfer surface pressure for HAP particles (π = 20 mN/m) has been determined experimentally.

**Figure 2:**
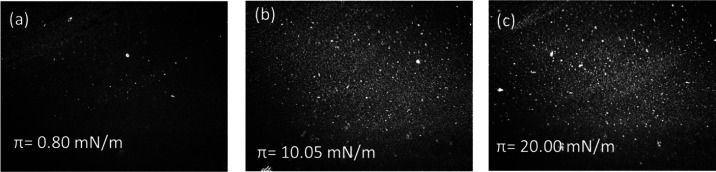
BAM images of the HAP monolayer at the air/water interface captured during the compression at π = 0.80 mN/m (a), π = 10.05 mN/m (b), and π = 20.00 mN/m (c).

The density of the film formed at the air/water interface and HAP aggregation were further investigated using BAM. The images captured during the compression at 0.80, 10.05, and 20.0 mN/m (Figure 2a–c) clearly demonstrate the changes in films’ morphology during the compression. The particles’ density increased, confirming the formation of a hydroxyapatite film at the air/water interface; yet, some HAP aggregates are visible in agreement with the data shown in Figure 1a. The aggregation results from the attractive interactions between HAP particles, causing an incomplete reversion.

**Figure 3:**
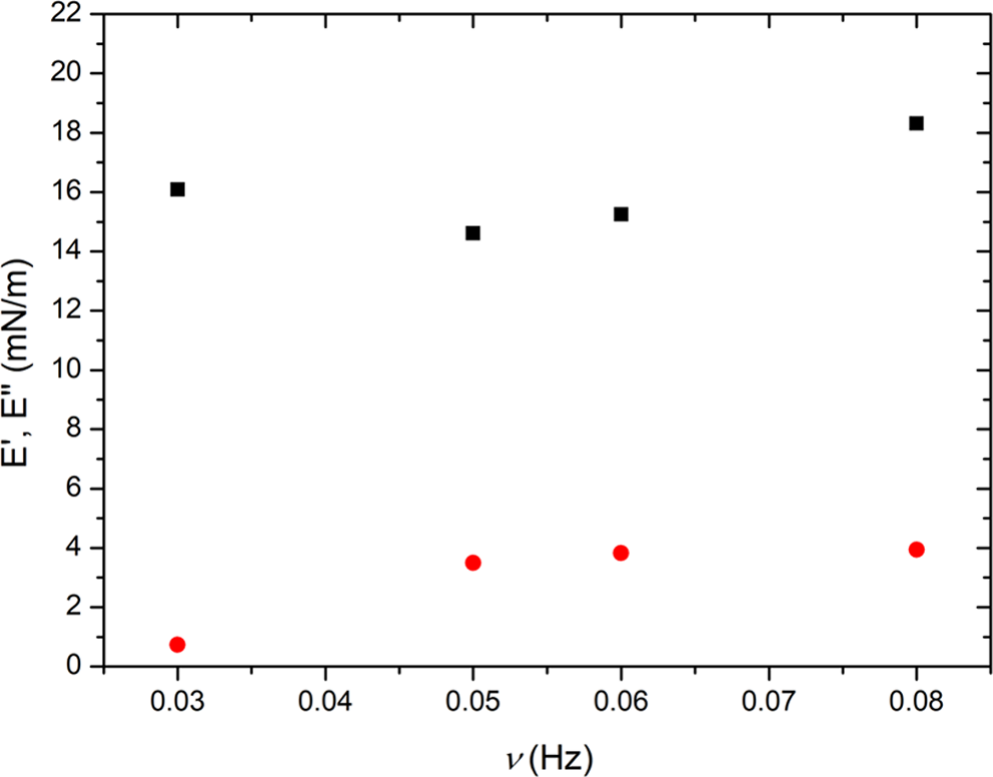
Elastic (red solid circle) and viscous (■) moduli were determined in the oscillating barrier experiment for the HAP film spread at the air/water interface and compressed to π= 20 mN/m.

In order to get insights into the stability of the HAP film subjected to compression, we performed the oscillating barrier experiment at π = 20 mN/m. The frequency dependence of elastic and viscous moduli for the HAP film is reported in Figure 3. In the whole frequency range, a visible domination of the elastic response over the viscous behavior, with the *E*′ values higher than *E*″, was noted. Similar values of dilational moduli were obtained earlier for the expanded mixed films containing silica nanoparticles.^38,50^ Therefore, the results presented in Figure 3 could be affected by the low density of the HAP film at 20 mN/m. Another contribution to surface dilational moduli is made by the rigid nature of nanoparticles. In addition, relatively low values of *E*′ and E” might be explained by the small strain applied (2%) within the oscillating barrier experiment. A similar effect was indicated in rheological studies on a poly(vinyl acetate) monolayer.^51^

**Figure 4:**
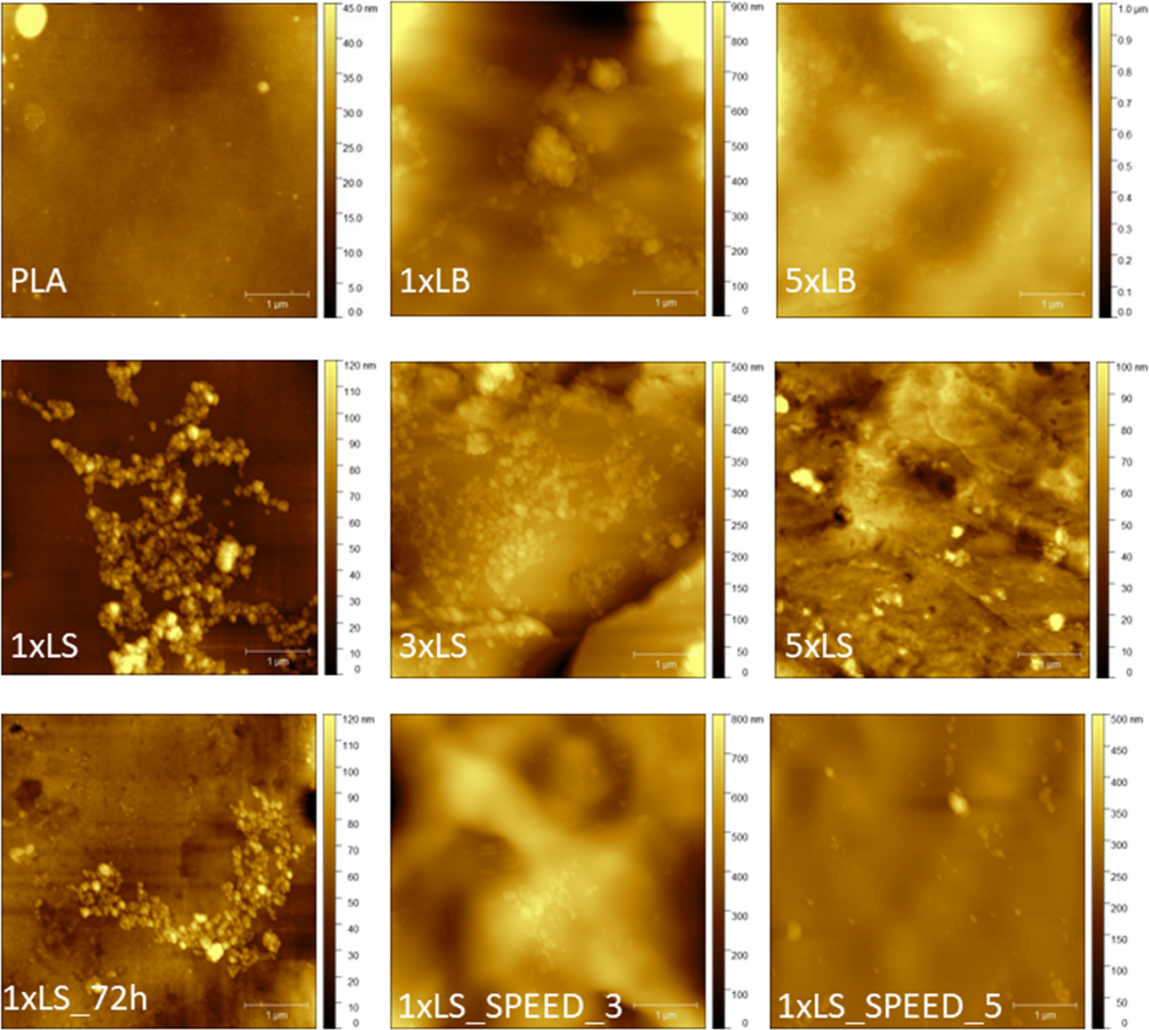
AFM topography images of PLA samples: PLA-pure poly(lactic acid), 1xLB-PLA coated with a single LB film, 5xLB-PLA coated with 5 LB layers, 1xLS-PLA coated with a single LS layer, 3xLS-PLA coated with 3 LS layers, 1xLS_72h-PLA coated with a single LS layer after 72 h, 1xLS_SPEED_3-PLA coated with a single LS layer at a deposition speed of 3 mm/min, and 1xLS_SPEED_5-PLA coated with a single LS layer at a deposition speed of 5 mm/min. The scanned area is 5 μm × 5 μm.

Langmuir monolayer was transferred onto the target substrate, i.e., 3D-printed PLA. Two types of deposition were applied: vertical (LB) and horizontal (LS). For both approaches, single and multiple layers were deposited. In the case of the LS method, various transfer speeds were also tested. The transfer ratios for all samples are shown in Table 1. The topography images gathered in the AFM measurements are shown in Figure 4 in addition to the quantitative AFM data listed in Table 2. The TR values for the first layer are much higher for LB films than for LS deposition. The TR value higher than unity means that the deposited material exists as a multilayer on the surface of PLA. On the other hand, the TR values lower than unity for all samples modified via the LS approach mean that less than 100% of the available monolayer material was deposited on PLA. This result can be explained by the aggregation of HAP particles facilitated during the LB transfer. The AFM data for 1xLB and 1xLS support the conclusion, since the maximum height of 1xLB and 5xLB is much higher than those for all samples coated with the LS film. In the case of images captured for 1xLS and 3xLS, one can distinguish clearly round-shape objects, which are not observed for 1xLB or 5xLB. Both mean and RMS roughness are much higher for Langmuir–Blodgett films than those for 1, 3, or 5 layers of Langmuir–Schaefer films. The TR values shown in Table 1 are not directly correlated with the surface coverage presented in Table 2. However, both parameters should be interpreted together. Despite similar surface coverage values obtained for 1xLB and 1xLS (20.15 and 26.20%, respectively), the structures of both films are different, which results from the TR values affected by the aggregation of HAP during the LB deposition.

**Table 1:** Transfer Ratios for LB and LS Depositions of HAP on PLA^a^

	transfer ratio for the deposition no.				
sample	**1**	**2**	**3**	**4**	**5**
1xLB	2.638	n.a.	n.a.	n.a.	n.a.
5xLB	2.987	0.531	1.041	–0.139	0.850
1xLS	0.729	n.a.	n.a.	n.a.	n.a.
3xLS	0.721	0.253	0.305	n.a.	n.a.
5xLS	0.674	0.398	0.272	0.276	0.309
1xLS_SPEED_3	0.189	n.a.	n.a.	n.a.	n.a.
1xLS_SPEED_5	0.029	n.a.	n.a.	n.a.	n.a.

a The names of the samples correspond to the type of deposition (LS or LB), the number of deposited layers (1, 3, or 5), and the deposition speed (3 or 5 mm/min).

**Table 2:** The Results of the Quantitative Analysis of AFM Data Obtained for PLA Samples (the Scanned Area is 5 μm × 5 μm)^a^

sample	mean roughness (*S*_a_) (nm)	RMS roughness (*S*_q_) (nm)	skewness (*S*_sk_)	excess kurtosis (S_ku_)	maximum height (*S*_*z*_) (nm)	surface coverage (%)
PLA	2.33	4.15	4.04	32.03	68.31	n.a.
1xLB	100.86	156.83	0.68	4.04	1349.55	20.15
5xLB	87.028	117.19	–0.99	4.38	1052.48	19.48
1xLS	12.10	16.57	1.99	4.64	144.98	26.20
3xLS	49.07	64.45	–0.34	1.47	591.56	49.81
5xLS	9.73	13.13	0.67	3.20	158.65	
1xLS_72h	9.15	13.08	0.56	4.82	165.16	12.41
1xLS_SPEED_3	97.49	120.17	–0.36	–0.10	768.87	6.57
1xLS_SPEED_5	59.23	73.323	–0.15	–0.01	503.10	5.76

a The names of the samples correspond to the images in Figure 4.

Comparing the surface coverage of 1 and 5 LB layers, one can observe that those values are almost the same, meaning that the deposition of multiple layers leads to the accumulation of the HAP particles on the surface of existing particles instead of lateral growth. In the case of the LS method, the surface coverage after the first transfer is 26.20%, but it reaches almost 50% after the deposition of 3 layers. Unfortunately, the determination of the surface coverage for 5 LS layers was not possible due to the difficulty in distinguishing the surface of PLA from HAP particles. Comparing the TR values for 5xLB and 5xLS leads to the conclusion that more material is deposited on the PLA surface via the LB procedure, but more homogeneous distribution is observed for the LS approach. Therefore, the LS technique was chosen for further studies.

The deposition speed was optimized based on the results of three independent transfers of the LS film: at 1 (1xLS), 3 (1xLS_SPEED_3), and 5 mm/min (1xLS_SPEED_5). The TR values in Table 1 clearly show that the best quality of the transfer was obtained for a withdrawal speed of 1 mm/min. Increasing the speed leads to a drastic decrease in the concentration of HAP particles on the surface. This effect might be associated with the critical deposition speed above which the meniscus formed on the water surface advances faster than the HAP particles can adsorb onto the substrate.^52^ This result is in agreement with the AFM data, indicating very low (below 7%) surface coverage for speeds higher than 1 mm/min. For these samples, the skewness and excess kurtosis are negative, which suggests more points below the average value in the height distribution curve and higher intensity of extreme values, respectively. These results indicate the presence of both small HAP particles and larger aggregates on the surface. Based on these results, the deposition speed of 1 mm/min was chosen for further transfers.

Moreover, the reproducibility of LS transfer was satisfactory, as evidenced by the TR values for the first layers for 1xLS, 3xLS, and 5xLS and the TR for another sample of PLA coated by a single LS film and shown in Figure S2 in the SI. The surface coverage for these two independent samples of 1xLS was 26.20 and 35.40%. These results as well as other data in Table 2 might be affected by the polydispersity of HAP particles.

**Figure 5:**
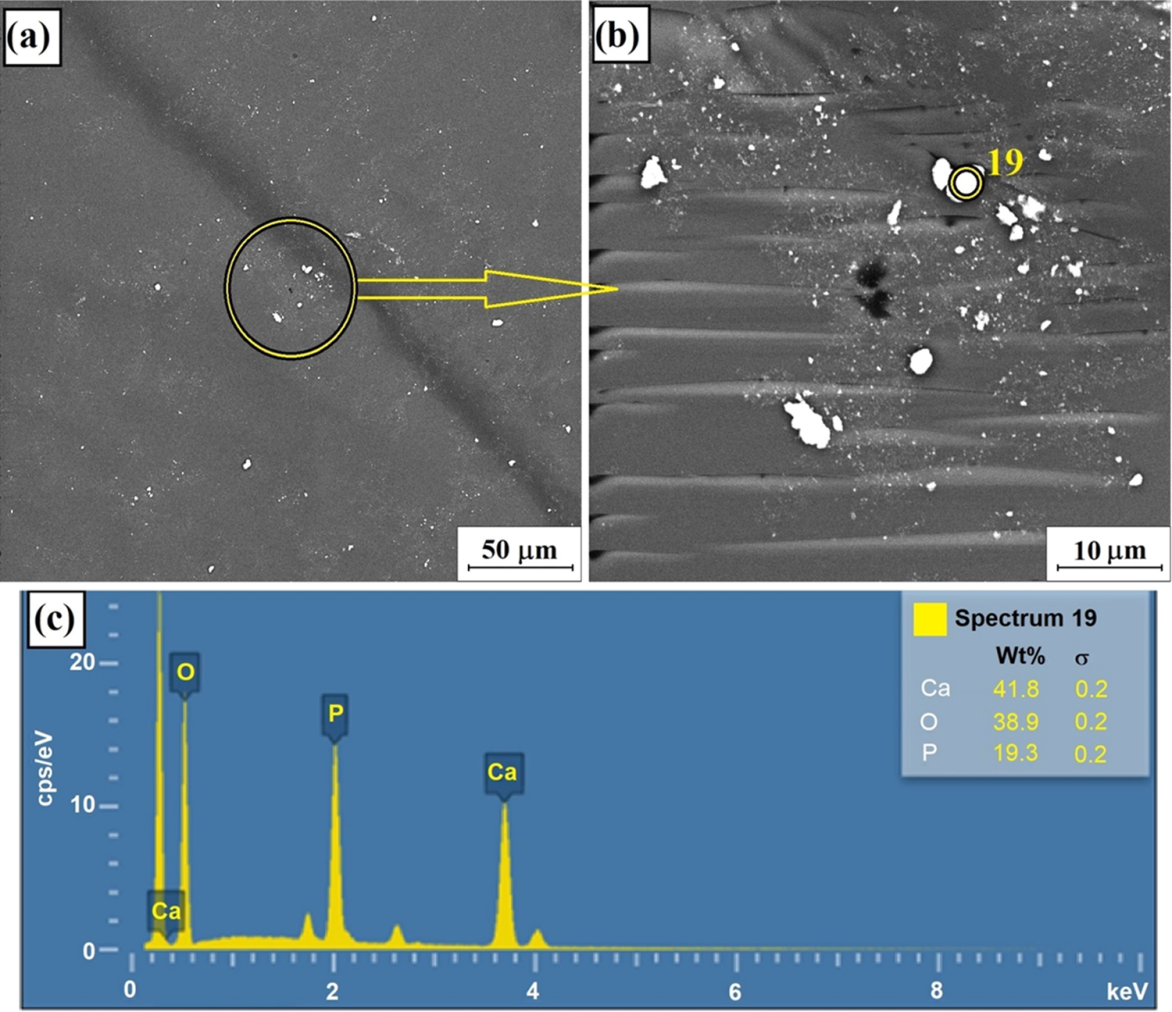
SEM image of HAP particles on the surface of PLA for two various magnifications (a, b) and the spectrum showing the Ca/O/P ratio for the selected point (c).

The stability of the coating over time was investigated for a single Langmuir–Schaefer deposition. The AFM images of that sample just after the preparation and 3 days later are shown in Figure 5 and are named 1xLS and 1xLS_72h, respectively. The topography of both samples does not show meaningful differences. The roughness parameters are slightly smaller after 72 h. The surface coverage is smaller for 1xLS_72h, which results from weak physical forces between HAP and PLA. It must be remembered that both images were gathered for two separate areas on the sample, which might affect the value of surface coverage. Moreover, the stability of the particles in the air environment may not be an indicator of their stability in the human body. Even though the loss of HAP particles after 24 h was as high as 50% according to the values in Table 2, the particles deposited on the implant could still affect the initial stage of osteointegration, which starts within a few minutes after the surgery. The presence of hydroxyapatite on the surface of bone implants may also decrease the risk of early implant-related infection resulting from biofilm formation, which is a serious challenge in orthopedic surgery.^53^ Taking into account the growing problem of antibiotic resistance,^54^ our approach might be an alternative strategy for modulating bacterial colonization and inflammatory reactions.

Comparing the roughness of the modified substrates and pure PLA, one can observe the increase of *S*_a_ and *S*_q_ after coating the material with HAP via the LB or LS method. This result may have an impact on protein adsorption, which induces cell attachment on the surface of a biomaterial.^55^ Therefore, an increased roughness (between 1 and 2 μm) is usually required in metallic implants.^56^ Nanoscale roughness produced by HAP particles on PLA is more difficult to control, but the nanometer-sized surface irregularities may influence the tissue response on a biomaterial, similar to the behavior of a titanium implant.^55^

The maximum height for 1xLS representing a single “monolayer” of HAP particles is 144.98 nm, which is smaller than that resulting from the particle size measurement. These discrepancies might be explained either by the tendency of the particles to adapt a disk shape on a solid support^57^ or by the nonuniform shape of the particles. The latter was further confirmed by the AFM using atomically flat mica as a solid substrate (see topography image in Figure S3 in the SI).

The HAP film transferred via single LS deposition onto the surface of a 3D-printed poly(lactic acid) sample was visualized by a scanning electron microscope and presented in Figure 5. The micrograph indicates a smooth polylactide surface with characteristic paths created upon filament stacking, as expected. Additionally, a number of whitish inclusions distributed throughout the PLA surface were noted, suggesting the presence of HAP particles. Their sizes vary significantly from 0.31 to 2.09 μm, as shown in Figure S4, confirming partial agglomeration of the particles. EDS analysis (Figure 5c) was employed to confirm the chemical composition of the particles detected on the surface. This method allows us to verify the calcium/phosphorus (Ca/P) mass ratio of the particles, bearing in mind a theoretical ratio value of hydroxyapatite of 1.67.^57^ The analysis of the EDS data proved that the Ca/P ratio equals 2.12, which might be explained by the phase purity of HAP, which specifically can be affected by the presence of calcium oxide.^58^ Hence, the presence of HAP particles on the PLA surface was confirmed.

Tuning the wettability of biomaterials is often desired to control osteoblast adhesion, especially in the case of bone-repairing compositions.^9,59^ According to recent studies, the material should be hydrophilic enough to achieve cell–implant adhesion and still hydrophobic enough to ensure cell–cell cohesion.^60^ Therefore, the surfaces of pure 3D-printed PLA and after a single layer deposition via LB and LS methods were characterized by water contact angle measurement (WCA) with the results shown in Table 3. The WCA value for pure PLA of 58° was noted. The value significantly varies from the one reported in our previous paper^1^ due to different preparation routes of the specimens, compression molded versus 3D-printed samples, which significantly influences their surface topography. The deposition of HAP particles on the PLA surface, both through LB or LS approach, did not induce further notable changes in WCA values, almost falling within the range of the observational error. This outcome can be explained by the inhomogeneous coverage of the substrates by the HAP particles. Nevertheless, the measured WCA values lie within the range acceptable for polymeric biomaterials.^60^ The similarities in the wettability of the 3D-printed PLA and HAP particles may also explain the physical adherence of HAP particles to the surface of the polymer during the transfer.

**Table 3:** Contact Angle Values Measured for Pure 3D-Printed PLA and PLA Modified via Deposition of a Single LB or LS Film

material	contact angle (deg)
PLA	58.10 ± 3.68
1xLS	68.92 ± 1.07
1xLB	63.08 ± 2.02

## Conclusions

Our study revealed that hydroxyapatite particles dispersed in ethanol can be spread at the air/water interface, compressed to relatively high surface pressure, and deposited on the surface of PLA. The HAP particles have been used earlier to modify the surface of titanium or stainless steel using electrophoretic deposition,^61^ plasma spray,^62^ sputtering process,^63^ and sol–gel or laser-assisted technique.^64^ In the case of poly(lactic acid), most of the modification methods focus on blending HAP particles with the polymer. The approach presented here is the first to report the surface modification of PLA with HAP via LB and LS methods. Compared to the above-mentioned strategies, the main advantages of our approach are quick procedure, small thickness of the HAP film, simplicity, and low cost in terms of material consumption. Moreover, the LB/LS technique allows the deposition of smaller particles, which are known as favorable for increased cell adhesion. Coating the PLA surface with small HAP particles via the LS approach alters the nanoscale roughness of the implant, which might be helpful in controlling its topography at the microscopic level.

Both LB and LS deposition can be employed to transfer HAP particles on a solid material; however, the quality of the transfer is significantly better for the Langmuir–Schafer approach. A single LS transfer provides larger surface coverage than the LB method, but the best effect was achieved by deposition of 3 LS layers. The difference between LB and LS transfer may be the effect of facilitated aggregation of HAP during the LB procedure, which forces higher mobility of the particles than the LS approach.

Even though the transferred LS film is not homogeneous, the obtained contact angle values of the PLA surface fall within the intended range and are adequate for biomedical applications. Further studies will focus on improving the adhesion of HAP nanoparticles to PLA and mixing HAP within an organic template by using amphiphilic biomolecules to create the coating that will tune the biological response of the host body to polymer bone implants. Furthermore, deposition of the HAP particles on the PLA surface together with the loading polymer with HAP, as shown in our previous work,^1^ might be a promising strategy to improve early-stage and long-term osteointegration.

## Supporting Information Available

Compression modulus plot; additional AFM images; transfer ratios; and SEM image (PDF)

## References

[ref1] Targonska S.; Dobrzynska-Mizera M.; Wujczyk M.; Rewak-Soroczynska J.; Knitter M.; Dopierala K.; Andrzejewski J.; Wiglusz R. J. New Way to Obtain the Poly(L-Lactide-Co-D,L-Lactide) Blend Filled with Nanohydroxyapatite as Biomaterial for 3D-Printed Bone-Reconstruction Implants. Eur. Polym. J. 2022, 165, 110997 10.1016/j.eurpolymj.2022.110997.

[ref2] Hickey D. J.; Ercan B.; Sun L.; Webster T. J. Adding MgO Nanoparticles to Hydroxyapatite–PLLA Nanocomposites for Improved Bone Tissue Engineering Applications. Acta Biomater. 2015, 14, 175–184. 10.1016/j.actbio.2014.12.004. 25523875

[ref3] Peng X.; Zhang Y.; Chen Y.; Li S.; He B. Synthesis and Crystallization of Well-Defined Biodegradable Miktoarm Star PEG-PCL-PLLA Copolymer. Mater. Lett. 2016, 171, 83–86. 10.1016/j.matlet.2016.02.057.

[ref4] Niu X.; Feng Q.; Wang M.; Guo X.; Zheng Q. Porous Nano-HA/Collagen/PLLA Scaffold Containing Chitosan Microspheres for Controlled Delivery of Synthetic Peptide Derived from BMP-2. J. Controlled Release 2009, 134 (2), 111–117. 10.1016/j.jconrel.2008.11.020. 19100794

[ref5] Mohamed K. R.; Beherei H. H.; El-Rashidy Z. M. In Vitro Study of Nano-Hydroxyapatite/Chitosan–Gelatin Composites for Bio-Applications. J. Adv. Res. 2014, 5 (2), 201–208. 10.1016/j.jare.2013.02.004. 25685488 PMC4294712

[ref6] Persson M.; Lorite G. S.; Kokkonen H. E.; Cho S.-W.; Lehenkari P. P.; Skrifvars M.; Tuukkanen J. Effect of Bioactive Extruded PLA/HA Composite Films on Focal Adhesion Formation of Preosteoblastic Cells. Colloids Surf., B 2014, 121, 409–416. 10.1016/j.colsurfb.2014.06.029. 24986753

[ref7] Senatov F. S.; Niaza K. V.; Zadorozhnyy M. Yu.; Maksimkin A. V.; Kaloshkin S. D.; Estrin Y. Z. Mechanical Properties and Shape Memory Effect of 3D-Printed PLA-Based Porous Scaffolds. J. Mech. Behav. Biomed. Mater. 2016, 57, 139–148. 10.1016/j.jmbbm.2015.11.036. 26710259

[ref8] Menzies K. L.; Jones L. The Impact of Contact Angle on the Biocompatibility of Biomaterials. Optom. Vision Sci. 2010, 87, 387–399. 10.1097/OPX.0b013e3181da863e. 20375749

[ref9] Gentleman M. M.; Gentleman E. The Role of Surface Free Energy in Osteoblast-Biomaterial Interactions. Int. Mater. Rev. 2014, 59 (8), 417–429. 10.1179/1743280414Y.0000000038.

[ref10] Jordá-Vilaplana A.; Fombuena V.; García-García D.; Samper M. D.; Sánchez-Nácher L. Surface Modification of Polylactic Acid (PLA) by Air Atmospheric Plasma Treatment. Eur. Polym. J. 2014, 58, 23–33. 10.1016/j.eurpolymj.2014.06.002.

[ref11] Laput O. A.; Vasenina I. V.; Botvin V. V.; Kurzina I. A. Surface Modification of Polylactic Acid by Ion, Electron Beams and Low-Temperature Plasma: A Review. J. Mater. Sci. 2022, 57 (4), 2335–2361. 10.1007/s10853-021-06687-3.

[ref12] Rasal R. M.; Janorkar A. V.; Hirt D. E. Poly(Lactic Acid) Modifications. Prog. Polym. Sci. 2010, 35 (3), 338–356. 10.1016/j.progpolymsci.2009.12.003.

[ref13] Oliveira O. N.; Caseli L.; Ariga K. The Past and the Future of Langmuir and Langmuir-Blodgett Films. Chem. Rev. 2022, 6459–6513. 10.1021/acs.chemrev.1c00754. 35113523

[ref14] Guney A.; Kara F.; Ozgen O.; Aksoy E. A.; Hasirci V.; Hasirci N. Surface Modification of Polymeric Biomaterials. In Biomaterials Surface Science; Wiley-VCH Verlag, 2013; pp 89–158.

[ref15] Prabhawathi V.; Boobalan T.; Sivakumar P. M.; Doble M. Antibiofilm Properties of Interfacially Active Lipase Immobilized Porous Polycaprolactam Prepared by LB Technique. PLoS One 2014, 9 (5), e96152 10.1371/journal.pone.0096152. 24798482 PMC4010425

[ref16] Bodik M.; Jergel M.; Majkova E.; Siffalovic P. Langmuir Films of Low-Dimensional Nanomaterials. Adv. Colloid Interface Sci. 2020, 283, 102239 10.1016/j.cis.2020.102239. 32854017

[ref17] Schultz D. G.; Lin X.; Li D.; Gebhardt J.; Meron M.; Viccaro P. J.; Lin B. Structure, Wrinkling, and Reversibility of Langmuir Monolayers of Gold Nanoparticles. J. Phys. Chem. A 2006, 110, 24522–24529. 10.1021/jp063820s. 17134211

[ref18] Ábrahám N.; Sebodouble acutek D.; Papp S.; Kodouble acuterösi L.; Dékány I. Two-Dimensional Arrangement of Monodisperse ZnO Particles with Langmuir-Blodgett Technique. Colloids Surf., A 2011, 384 (1–3), 80–89. 10.1016/j.colsurfa.2011.03.025.

[ref19] Paczesny J.; Wolska-Pietkiewicz M.; Binkiewicz I.; Janczuk-Richter M. Langmuir and Langmuir Blodgett Films of Zinc Oxide (ZnO) Nanocrystals Coated with Polyhedral Oligomeric Silsesquioxanes (POSS). J. Colloid Interface Sci. 2021, 600, 784–793. 10.1016/j.jcis.2021.05.085. 34051466

[ref20] Clemente-León M.; Coronado E.; López-Muñoz Á.; Repetto D.; Mingotaud C.; Brinzei D.; Catala L.; Mallah T. Magnetic Langmuir–Blodgett Films of Bimetallic Coordination Nanoparticles of Cs0.4Ni[Cr(CN)6]0.9. Chem. Mater. 2008, 20 (14), 4642–4652. 10.1021/cm8006765.

[ref21] Burhan Mohamed K.; Ghaderi S.; Hallaj R.; Hassanzadeh A. Langmuir–Blodgett Films of Magnetic Nanowires. Mater. Sci. Eng., B 2023, 296, 116649 10.1016/j.mseb.2023.116649.

[ref22] Fujii S.; Morita T.; Kimura S. Fabrication of Langmuir–Blodgett Film of a Fullerene Derivative with a Cyclic Peptide as an Anchor. Bioconjugate Chem. 2007, 18 (6), 1855–1859. 10.1021/bc700159c. 17941685

[ref23] Black A.; Roberts J.; Acebrón M.; Bernardo-Gavito R.; Alsharif G.; J Urbanos F.; H Juárez B.; V Kolosov O.; J Robinson B.; Miranda R.; L Vázquez de Parga A.; Granados D.; J Young R. Large-Area Heterostructures from Graphene and Encapsulated Colloidal Quantum Dots via the Langmuir–Blodgett Method. ACS Appl. Mater. Interfaces 2018, 10 (8), 6805–6809. 10.1021/acsami.7b17102. 29436818

[ref24] Nie Y.; Tian M.; Zhang Q.; Wang L.; Ji Z.; Chen X.; Yang S.; Song W.; Wu C.; Xu H.; Cao M. Controlled Fabrication of Biocompatible Graphene Oxide Langmuir–Blodgett Films by Size and Surface Property Manipulation. J. Dispers Sci. Technol. 2022, 43 (12), 1747–1754. 10.1080/01932691.2021.1880430.

[ref25] Ariga K. Liquid Interfacial Nanoarchitectonics: Molecular Machines, Organic Semiconductors, Nanocarbons, Stem Cells, and Others. Curr. Opin. Colloid Interface Sci. 2023, 63, 101656 10.1016/j.cocis.2022.101656.

[ref26] Fainerman V. B.; Aksenenko E. V.; Krägel J.; Miller R. Thermodynamics, Interfacial Pressure Isotherms and Dilational Rheology of Mixed Protein-Surfactant Adsorption Layers. Adv. Colloid Interface Sci. 2016, 200–222. 10.1016/j.cis.2015.06.004. 26198014

[ref27] Dopierała K.; Krajewska M.; Weiss M. Physicochemical Characterization of Oleanolic Acid-Human Serum Albumin Complexes for Pharmaceutical and Biosensing Applications. Langmuir 2020, 36 (13), 3611–3623. 10.1021/acs.langmuir.0c00087. 32176505

[ref28] Skrzypiec M.; Weiss M.; Dopierała K.; Prochaska K. Langmuir-Blodgett Films of Membrane Lipid in the Presence of Hybrid Silsesquioxane, a Promising Component of Biomaterials. Mater. Sci. Eng., C 2019, 105, 110090 10.1016/j.msec.2019.110090. 31546436

[ref29] Dopierała K.; Krajewska M.; Prochaska K. Study on PH-Dependent Interactions of Linoleic Acid with α-Lactalbumin. Food Hydrocolloids 2021, 111, 106217 10.1016/j.foodhyd.2020.106217.

[ref30] Dopierała K.; Weiss M.; Krajewska M.; Błońska J. Towards Understanding the Binding Affinity of Lipid Drug Carriers to Serum Albumin. Chem. Phys. Lipids 2023, 250, 105271 10.1016/j.chemphyslip.2022.105271. 36509110

[ref31] Wydro P.; Krajewska B.; Hac-Wydro K. Chitosan as a Lipid Binder: A Langmuir Monolayer Study of Chitosan-Lipid Interactions. Biomacromolecules 2007, 8 (8), 2611–2617. 10.1021/bm700453x. 17630796

[ref32] Ruiz G. C. M.; Cruz M. A. E.; Faria A. N.; Zancanela D. C.; Ciancaglini P.; Ramos A. P. Biomimetic Collagen/Phospholipid Coatings Improve Formation of Hydroxyapatite Nanoparticles on Titanium. Mater. Sci. Eng., C 2017, 77, 102–110. 10.1016/j.msec.2017.03.204. 28531974

[ref33] Broniatowski M.; Bojarski J.; Wydro P. Langmuir Monolayers as Models of the Lipid Matrix of Cyanobacterial Thylakoid Membranes. J. Mol. Liq. 2022, 368, 120727 10.1016/j.molliq.2022.120727.

[ref34] Broniatowski M.; Sobolewska K.; Flasiński M.; Wydro P. Studies on the Interactions of Bisphenols with Anionic Phospholipids of Decomposer Membranes in Model Systems. Biochim. Biophys. Acta, Biomembr. 2016, 1858 (4), 756–766. 10.1016/j.bbamem.2016.01.017. 26806160

[ref35] Lenhert S.; Meier M. B.; Meyer U.; Chi L.; Wiesmann H. P. Osteoblast Alignment, Elongation and Migration on Grooved Polystyrene Surfaces Patterned by Langmuir-Blodgett Lithography. Biomaterials 2005, 26 (5), 563–570. 10.1016/j.biomaterials.2004.02.068. 15276364

[ref36] de Faria A. N.; Cruz M. A. E.; Ruiz G. C. M.; Zancanela D. C.; Ciancaglini P.; Ramos A. P. Different Compact Hybrid Langmuir–Blodgett-Film Coatings Modify Biomineralization and the Ability of Osteoblasts to Grow. J. Biomed. Mater. Res., Part B 2018, 106 (7), 2524–2534. 10.1002/jbm.b.34069. 29314671

[ref37] Abdulla S.; Pullithadathil B. Unidirectional Langmuir–Blodgett-Mediated Alignment of Polyaniline-Functionalized Multiwalled Carbon Nanotubes for NH3 Gas Sensor Applications. Langmuir 2020, 36 (39), 11618–11628. 10.1021/acs.langmuir.0c02200. 32902997

[ref38] Dopierała K.; Bojakowska K.; Karasiewicz J.; Maciejewski H.; Prochaska K. Interfacial Behaviour of Cubic Silsesquioxane and Silica Nanoparticles in Langmuir and Langmuir-Blodgett Films. RSC Adv. 2016, 6 (97), 94934–94941. 10.1039/C6RA18255K.

[ref39] Dopierała K.; Kołodziejczak-Radzimska A.; Prochaska K.; Jesionowski T. Immobilization of Lipase in Langmuir – Blogett Film of Cubic Silsesquioxane on the Surface of Zirconium Dioxide. Appl. Surf. Sci. 2022, 573, 151184 10.1016/j.apsusc.2021.151184.

[ref40] de Souza Furtado F. A.; Caseli L. Enzyme Activity Preservation for Galactose Oxidase Immobilized in Stearic Acid Langmuir-Blodgett Films. Thin Solid Films 2020, 709, 138253 10.1016/j.tsf.2020.138253.

[ref41] Langmuir I.; Schaefer V. J. Activities of Urease and Pepsin Monolayers. J. Am. Chem. Soc. 1938, 60 (6), 1351–1360. 10.1021/ja01273a023.

[ref42] Bhuvanesh T.; Saretia S.; Roch T.; Schöne A.-C.; Rottke F. O.; Kratz K.; Wang W.; Ma N.; Schulz B.; Lendlein A. Langmuir-Schaefer Films of Fibronectin as Designed Biointerfaces for Culturing Stem Cells. Polym. Adv. Technol. 2017, 28 (10), 1305–1311. 10.1002/pat.3910.

[ref43] Calejo M. T.; Saari J.; Vuorenpää H.; Vuorimaa-Laukkanen E.; Kallio P.; Aalto-Setälä K.; Miettinen S.; Skottman H.; Kellomäki M.; Juuti-Uusitalo K. Co-Culture of Human Induced Pluripotent Stem Cell-Derived Retinal Pigment Epithelial Cells and Endothelial Cells on Double Collagen-Coated Honeycomb Films. Acta Biomater. 2020, 101, 327–343. 10.1016/j.actbio.2019.11.002. 31711900

[ref44] Liu S.; Bae M.; Hao L.; Oh J. K.; White A. R.; Min Y.; Cisneros-Zevallos L.; Akbulut M. Bacterial Antifouling Characteristics of Helicene—Graphene Films. Nanomaterials 2021, 11 (1), 89 10.3390/nano11010089. 33401616 PMC7830421

[ref45] Sołoducho J.; Cabaj J. Biocatalysts Immobilized in Ultrathin Ordered Films. Sensors 2010, 10 (11), 10298–10313. 10.3390/s101110298. 22163470 PMC3230983

[ref46] Ariga K. Don’t Forget Langmuir-Blodgett Films 2020: Interfacial Nanoarchitectonics with Molecules, Materials, and Living Objects. Langmuir 2020, 7158–7180. 10.1021/acs.langmuir.0c01044. 32501699

[ref47] Parchine M.; McGrath J.; Bardosova M.; E Pemble M. Large Area 2D and 3D Colloidal Photonic Crystals Fabricated by a Roll-to-Roll Langmuir–Blodgett Method. Langmuir 2016, 32 (23), 5862–5869. 10.1021/acs.langmuir.6b01242. 27218474

[ref48] Mendoza A. J.; Guzmán E.; Martínez-Pedrero F.; Ritacco H.; Rubio R. G.; Ortega F.; Starov V. M.; Miller R. Particle Laden Fluid Interfaces: Dynamics and Interfacial Rheology. Adv. Colloid Interface Sci. 2014, 206, 303–319. 10.1016/j.cis.2013.10.010. 24200090

[ref49] Schultz D. G.; Lin X.-M.; Li D.; Gebhardt J.; Meron M.; Viccaro J.; Lin B. Structure, Wrinkling, and Reversibility of Langmuir Monolayers of Gold Nanoparticles. J. Phys. Chem. B 2006, 110 (48), 24522–24529. 10.1021/jp063820s. 17134211

[ref50] Guzmán E.; Liggieri L.; Santini E.; Ferrari M.; Ravera F. Influence of Silica Nanoparticles on Dilational Rheology of DPPC-Palmitic Acid Langmuir Monolayers. Soft Matter 2012, 8 (14), 3938–3948. 10.1039/c2sm07097a.

[ref51] Morioka T.; Kawaguchi M. Surface Dilational Moduli of Polymer and Blended Polymer Monolayers Spread at Air-Water Interfaces. Adv. Colloid Interface Sci. 2014, 214, 1–16. 10.1016/j.cis.2014.10.003. 25456455

[ref52] Buhaenko M. R.; Richardson R. M. Measurements of the Forces of Emersion and Immersion and Contact Angles during Langmuir-Blodgett Deposition. Thin Solid Films 1988, 159 (1–2), 231–238. 10.1016/0040-6090(88)90634-7.

[ref53] Seebach E.; Kubatzky K. F. Chronic Implant-Related Bone Infections-Can Immune Modulation Be a Therapeutic Strategy?. Front. Immunol. 2019, 10, 1724 10.3389/fimmu.2019.01724. 31396229 PMC6664079

[ref54] Petchiappan A.; Chatterji D. Antibiotic Resistance: Current Perspectives. ACS Omega 2017, 2 (10), 7400–7409. 10.1021/acsomega.7b01368. 30023551 PMC6044581

[ref55] Gongadze E.; Kabaso D.; Bauer S.; Slivnik T.; Schmuki P.; van Rienen U.; Iglič A. Adhesion of Osteoblasts to a Nanorough Titanium Implant Surface. Int. J. Nanomed. 2011, 6, 1801–1816. 10.2147/IJN.S21755. PMC3173045 21931478

[ref56] Parithimarkalaignan S.; Padmanabhan T. V. Osseointegration: An Update. J. Indian Prosthodont Soc. 2013, 13 (1), 2–6. 10.1007/s13191-013-0252-z. 24431699 PMC3602536

[ref57] Miculescu F.; Luţă C.; Constantinescu A. E.; Maidaniuc A.; Mocanu A.-C.; Miculescu M.; Voicu Ş. I.; Ciocan L. T. Considerations and Influencing Parameters in EDS Microanalysis of Biogenic Hydroxyapatite. J. Funct. Biomater. 2020, 11 (4), 82 10.3390/jfb11040082. 33203117 PMC7711801

[ref58] Wang H.; Lee J.-K.; Moursi A.; Lannutti J. J. Ca/P Ratio Effects on the Degradation of Hydroxyapatitein Vitro. J. Biomed. Mater. Res. 2003, 67A (2), 599–608. 10.1002/jbm.a.10538. 14566803

[ref59] Shayan M.; Jung Y.; Huang P. S.; Moradi M.; Plakseychuk A. Y.; Lee J. K.; Shankar R.; Chun Y. Improved Osteoblast Response to UV-Irradiated PMMA/TiO2 Nanocomposites with Controllable Wettability. J. Mater. Sci.: Mater. Med. 2014, 25 (12), 2721–2730. 10.1007/s10856-014-5284-3. 25074833

[ref60] Tzoneva R.; Faucheux N.; Groth T. Wettability of Substrata Controls Cell–Substrate and Cell–Cell Adhesions. Biochim. Biophys. Acta, Gen. Subj. 2007, 1770 (11), 1538–1547. 10.1016/j.bbagen.2007.07.008. 17804166

[ref61] Zhang C.; Uchikoshi T.; Liu L.; Iwanami-Kadowaki K.; Uezono M.; Moriyama K.; Kikuchi M. Antibacterial-Functionalized Ag Loaded-Hydroxyapatite (HAp) Coatings Fabricated by Electrophoretic Deposition (EPD) Process. Mater. Lett. 2021, 297, 129955 10.1016/j.matlet.2021.129955.

[ref62] Chambard M.; Marsan O.; Charvillat C.; Grossin D.; Fort P.; Rey C.; Gitzhofer F.; Bertrand G. Effect of the Deposition Route on the Microstructure of Plasma-Sprayed Hydroxyapatite Coatings. Surf. Coat. Technol. 2019, 371, 68–77. 10.1016/j.surfcoat.2019.01.027.

[ref63] Yang Y.; Kim K.; Ong J. A Review on Calcium Phosphate Coatings Produced Using a Sputtering Process as an Alternative to Plasma Spraying. Biomaterials 2005, 26 (3), 327–337. 10.1016/j.biomaterials.2004.02.029. 15262475

[ref64] Duta L.; Oktar F. N.; Stan G. E.; Popescu-Pelin G.; Serban N.; Luculescu C.; Mihailescu I. N. Novel Doped Hydroxyapatite Thin Films Obtained by Pulsed Laser Deposition. Appl. Surf. Sci. 2013, 265, 41–49. 10.1016/j.apsusc.2012.10.077.

