# Supplementary material for: Surface Functionalization of Poly(lactic acid) via Deposition of Hydroxyapatite Monolayers for Biomedical Applications
Source: Langmuir. 2023 Oct 26;39(44):15610–9. doi: 10.1021/acs.langmuir.3c01914 (PMC10634356; doi:10.1021/acs.langmuir.3c01914)
Supplement: Supplementary file 1 — la3c01914_si_001.pdf [file la3c01914_si_001.pdf]

Surface functionalization of poly(lactic acid) *via* deposition of hydroxyapatite monolayers for biomedical applications

Katarzyna Dopierała[1], Monika Knitter[2], Monika Dobrzyńska-Mizera[2], Jacek Andrzejewski[2], Aneta Bartkowska[3], Krystyna Prochaska[1]

1- Institute of Chemical Technology and Engineering, Poznan University of Technology, Berdychowo 4, 60-965 Poznań, Poland

2- Institute of Material Technology, Poznan University of Technology, Piotrowo 3, 61-138 Poznan, Poland

3- Poznan University of Technology, Faculty of Materials Engineering and Technical Physics, Institute of Material Science and Engineering, Jana Pawła II 24, 61-138, Poznan, Poland

Supporting information

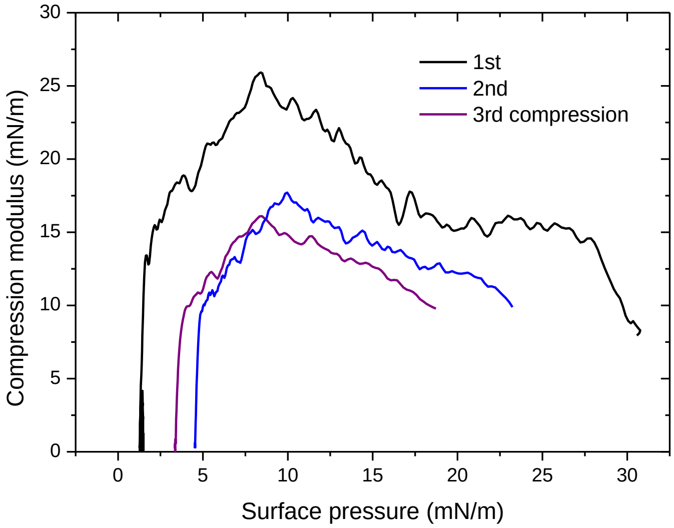

Figure S1 The compression modulus determined from the isotherm data using Eq.1

Table S1 Transfer ratios for 9 strokes of LB transfer of HAP on PLA which compared to the values of TR for 1xLB and 5xLB in main text demonstrate insufficient reproducibility of LB approach and loss of the material from the substrate at 6th and 8th deposition.

Layer no.	Transfer ratio
**1st**	1.068
**2nd**	0.152
**3rd**	0.697
**4th**	0.032
**5th**	0.577
**6th**	-0.122
**7th**	0.811
**8th**	-0.145
**9th**	0.716

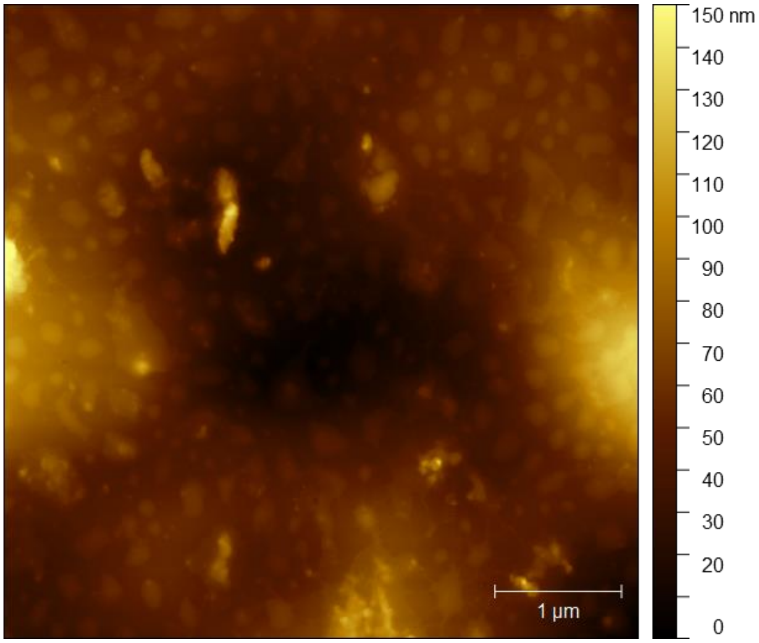

Figure S2 AFM image of PLA coated by HAP particles (1 layer, LS transfer), the scanned area is 5x5 µm. The surface coverage is 35.46%. The TR value is 0.690.

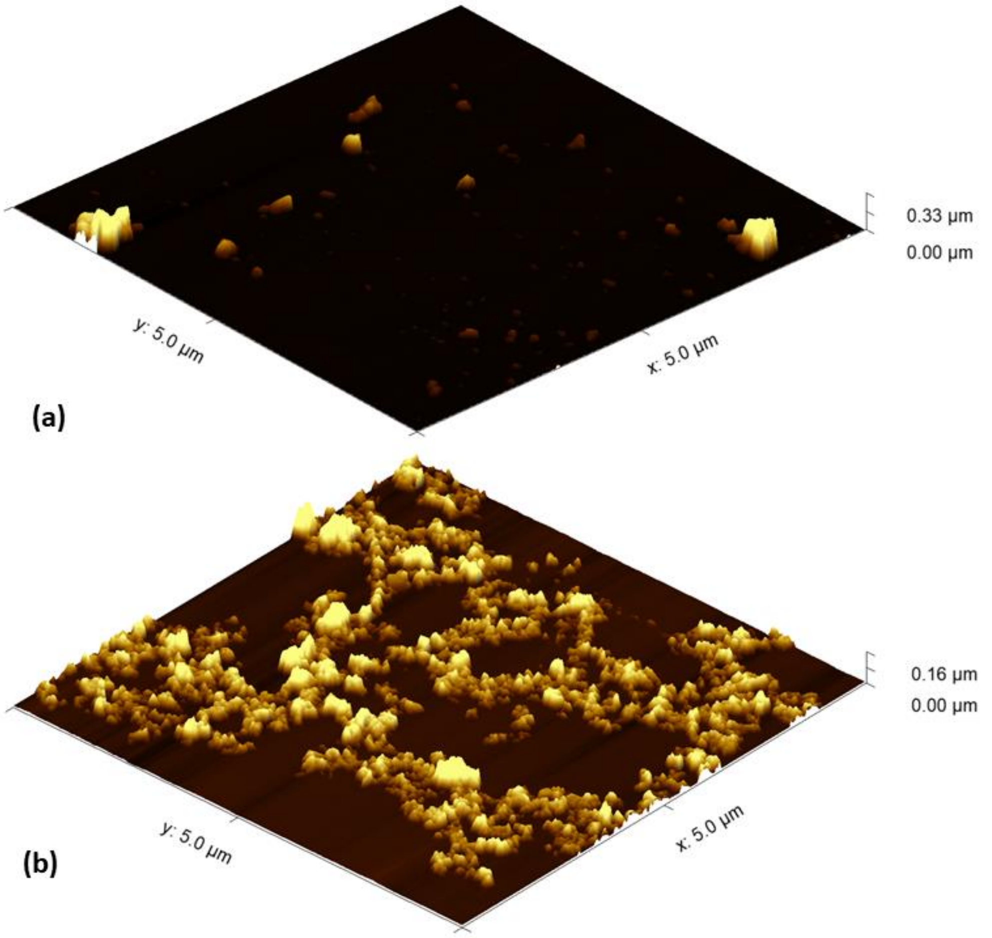

Figure S3 The topography of HAP film (images size 5x5μm), built of 1 (a) and (b) 5 layers of HAP deposited by Langmuir-Blodgett approach on mica. For the multilayer transfer positive TR were obtained exclusively for upstrokes: TR (1st 2.085, 2nd -0.67, 3rd 1.843, 4th -1.054, 5th 1.476); the surface coverage was approx. 14%. The substrates for the depositions were freshly cleaved mica sheets of AFM grade. The statistical quantities for Figure S2b are RMS roughness: 17.65 nm, mean roughness: 13.76 nm, skewness: 1.953, excess kurtosis: 4.578, and maximum height: 160 nm.

To evaluate the topography of HAP particles on a solid material, particularly the height of HAP particles without the impact of the structure of 3D printed PLA, the deposition was performed using atomically flat mica. Firstly, a single layer of HAP was deposited vertically with the topography image shown in Figure S2a. The surface coverage was very low due to insufficient transfer quality. Therefore, the deposition was repeated as a multilayer transfer. The topography image shown in Figure S3b suggests the significantly larger amount of HAP particles existing on the surface in comparison to the single transfer with confirmed the presence of aggregates. The

HAP particles were distributed throughout the surface reaching an approximate coverage of 14% as calculated from the topography image. The values of TR suggest that each upstroke is accompanied by the transfer of the particles from the interface onto the mica surface. However, during the downstrokes, a significant loss of the monolayer material was observed. Such behavior is often reported for LB films of typical amphiphiles as Z-type deposition[1]. The maximum height (160 nm) is in agreement with the thickness of the coating in 1xLS presented in Figure 4.

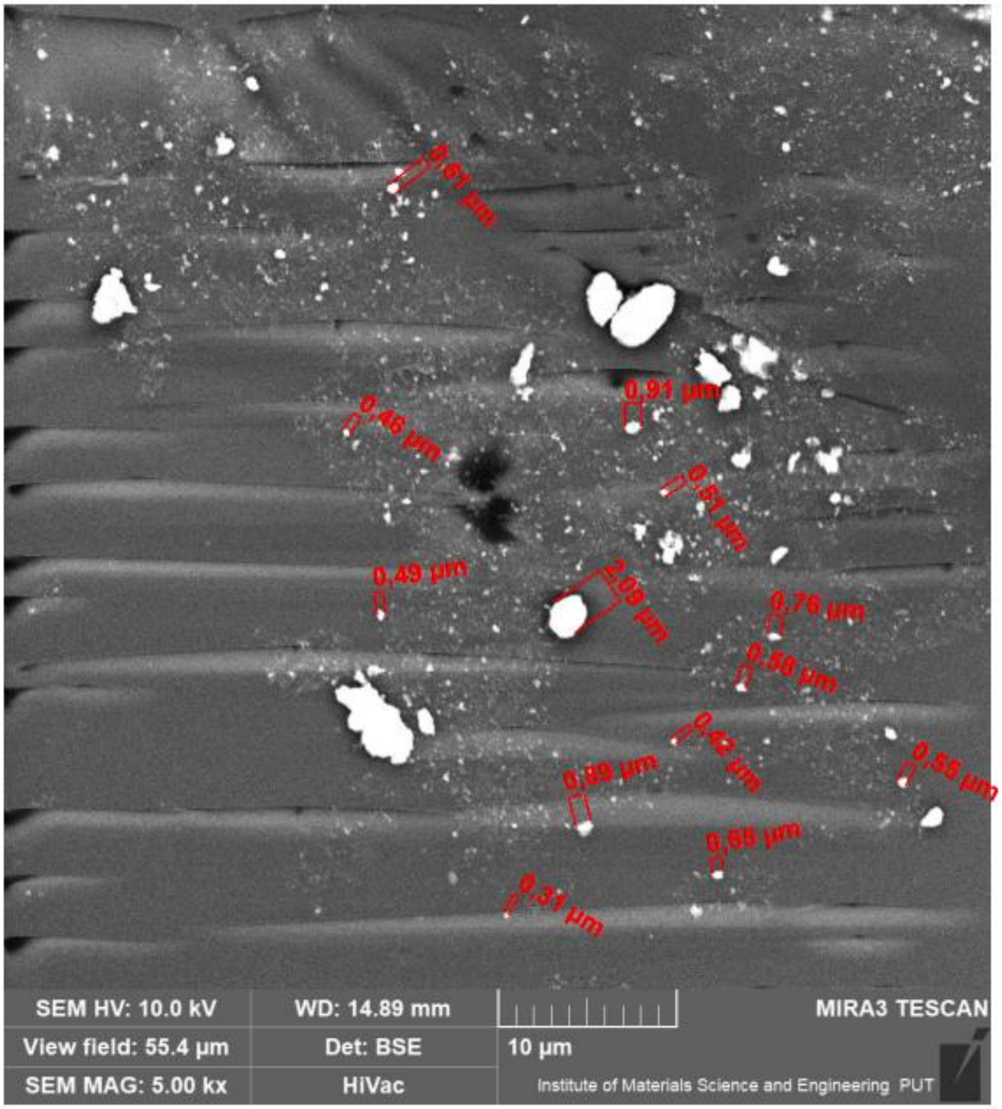

Figure S4. SEM image of PLA coated by HAP particles *via* LS method.

References

(1) Malhotra, B. D.; Singhal, R. Conducting Polymer Based Biomolecular Electronic Devices. *Pramana - Journal of Physics* **2003**, *61* (2), 331–343. https://doi.org/10.1007/BF02708313.
